# Research on the Method of Predicting Fractional Flow Reserve Based on Multiple Independent Risk Factors

**DOI:** 10.3389/fphys.2021.716877

**Published:** 2021-08-13

**Authors:** Honghui Zhang, Gaoyang Li, Qianwen Hou, Yinlong Yang, Hongge Wei, Yujia Yang, Zhuoran Qu, Jinjie Xie, Aike Qiao

**Affiliations:** ^1^Faculty of Environment and Life, Beijing University of Technology, Beijing, China; ^2^College of Engineering, Inner Mongolia University for Nationalities, Tongliao, China; ^3^Institute of Fluid Science, Tohoku University, Miyagi, Japan; ^4^Beijing Anzhen Hospital, Capital Medical University, Beijing, China

**Keywords:** fractional flow reserve, coronary stenosis, multiple independent risk factors, improved analytic hierarchy process (IAHP), coronary heart disease (CAD)

## Abstract

The use of diameter stenosis (DS), as revealed by coronary angiography, for predicting fractional flow reserve (FFR) usually results in a high error rate of detection. In this study, we investigated a method for predicting FFR in patients with coronary stenosis based on multiple independent risk factors. The aim of the study was to improve the accuracy of detection. First, we searched the existing literature to identify multiple independent risk factors and then calculated the corresponding odds ratios. The improved analytic hierarchy process (IAHP) was then used to determine the weighted value of each independent risk factor, based on the corresponding odds ratio. Next, we developed a novel method, based on the top seven independent risk factors with the highest weighted values, to predict FFR. This model was then used to predict the FFR of 253 patients with coronary stenosis, and the results were then compared with previous methods (DS alone and a simplified scoring system). In addition to DS, we identified a range of other independent risk factors, with the highest weighted values, for predicting FFR, including gender, body mass index, location of stenosis, type of coronary artery distribution, left ventricular ejection fraction, and left myocardial mass. The area under the receiver-operating characteristic curve (AUC) for the newly developed method was 84.3% (95% CI: 79.2–89.4%), which was larger than 65.3% (95% CI: 61.5–69.1%) of DS alone and 74.8% (95% CI: 68.4–81.2%) of the existing simplified scoring system. The newly developed method, based on multiple independent risk factors, effectively improves the prediction accuracy for FFR.

## Introduction

Coronary heart disease, including coronary stenosis, is associated with the highest mortality rate of diseases worldwide (Bruyne et al., 2014; Yang et al., 2017; Li et al., 2021). Many numerous studies have proved that fractional flow reserve (FFR) can effectively help to diagnose the severity of coronary stenosis and further assist in the proposal of treatment options (Pijls et al., 2007; Nørgaard et al., 2014; Lu et al., 2017). FFR is also known to significantly improve the prognosis of patients with percutaneous coronary intervention (PCI) with severe coronary stenosis (Tonino et al., 2010; Chinnaiyan et al., 2017; Caroline et al., 2018). Therefore, the accurate measurement and prediction of FFR has immense clinical significance for the treatment of patients with coronary stenosis, especially those with severe stenosis.

Currently, the prediction of FFR is mainly based on diameter stenosis (DS) (≥50%), as determined by coronary angiography (Bruyne et al., 2014; Nakamura et al., 2014). However, simulation studies and case reports have shown that the predictive accuracy is low when only DS is used as the criterion. In a previous study, Park et al. (2012) performed invasive FFR and coronary angiography on 1,129 lesions with coronary stenosis, and the predictive accuracy of FFR was 24.5% when using DS ≥ 50% as the main criterion. In another study, invasive FFR, based on DS coronary angiography, was performed on 200 patients with coronary stenosis, and the predictive accuracy of FFR was only 26% (Curzen et al., 2014). A slightly increased accuracy of FFR prediction (30.5%) was achieved in the work of Cho et al. (2014), who compared the results of invasive FFR and coronary angiography on 643 lesions with coronary stenosis and used DS as the only criterion. Based on DS of coronary angiography to predict FFR, especially angiography-derived FFR, it had some limitations. The angiography-derived FFR was invasive detection for patients and inconveniently derived for cardiologists (Tu et al., 2016; Westra et al., 2018). Geng et al. (2021) presented that angiography-derived FFR ignored the influence of individual risk factors of patients [such as gender, age, heart rate (HR), and so on] on predicting FFR. Based on the available literature, and the limitedly used DS of coronary angiography to predict FFR, there is a clear urgency to develop a new method to calculate FFR.

Some independent risk factors (such as gender and the length of coronary stenosis) have been confirmed to be strongly correlated with FFR (Kim et al., 2012; Westergren et al., 2017). These independent risk factors have been applied to the prediction of FFR and showed clear improvements in predictive accuracy. Natsumeda et al. (2015) derived a scoring system based on DS, the extent of stenosis, bifurcation, and the length of stenosis and managed to increase the prediction accuracy by 11.8% when compared to the application of DS and coronary angiography as the only criterion. In another study, Wong et al. (2015) proposed the DILEMMA score for the prediction of FFR; this involved DS, the length of stenosis, and the myocardial jeopardy index and improved the predictive accuracy by 22.2% when compared with a method that was based only on DS. Subsequently, Matar et al. (2016) developed a predictive method for FFR that was based on gender, DS, the length of stenosis, and the structure of the left anterior descending (LAD), and the resulting predictive accuracy of this method reached 73.5%. However, these studies incorporated a limited number of risk factors (<4). In addition, the relative weighting of each of the factors used in these studies was derived from the qualitative perspective of empirical judgment of clinicians. The predictive accuracy of these studies was insufficient to be applied by angiographers when compared to the method described by Taylor (84.3%) (Koo et al., 2011). Therefore, it is necessary to develop a new predictive method for FFR that integrates multiple independent risk factors and quantitatively calculates the corresponding weightings, thus improving the predictive accuracy for FFR.

In this study, we screened the existing literature and applied the improved analytic hierarchy process (IAHP) to identify multiple independent risk factors. We calculated the corresponding weightings for these factors and developed a new method for predicting FFR, which was subsequently validated using 253 clinical cases that underwent invasive FFR and coronary angiography.

## Methods

### Constructing a Hierarchical Structure for the Prediction of FFR

First, we screened the existing literature to identify the independent risk factors for predicting FFR in the coronary artery. Our retrieval strategy included scanning two databases (PubMed and Web of Science) for a series of query terms (coronary hemodynamic, or FFR and risk factors) in articles published up to December 2020. Next, two investigators independently screened the abstracts, titles, and full text (when appropriate). Then, they assessed the quality of the studies using the Newcastle–Ottawa Scale (NOS), including selection (0–4 points), comparability (0–2 points), and exposure (0–3 points), as described previously (Pham et al., 2019; Tian et al., 2020). We focused on the literature where the number of evaluation points was 6 or more and included the odds ratios of the independent risk factors. Next, we constructed a hierarchical structure for predicting FFR, including target, intermediate, and variable layers.

Based on the odds ratio, we used the IAHP and hierarchical structure to determine the relative weighting of each element. To do this, we first constructed an importance degree matrix, solved the feature vector and the maximum characteristic root of the matrix, evaluated the consistency of the odds ratio and importance degree of each element, and finally calculated the total weighting (Kayet et al., 2018; Han et al., 2020).

#### Constructing the Importance Degree Matrix

The importance degree matrix, showing a comparison between every two elements at the intermediate layer and the variable layer, was derived from the odds ratios of each element. An importance degree was assigned in the importance degree matrix by comparing every two elements; this ranged from 1 (equally important) to 9 (extremely important), such that the importance degree was more than 0, or 1/9 (equally important) to 1 (extremely important) in the opposite case (Kharat et al., 2019; Mokarram et al., 2020).

If *A*_*ij*_ represents the importance degree for comparing the element *i* with element *j* (*i, j* =1, 2, 3, 4, 5, 6) in the importance degree matrix, then *i* and *j* represent the coordinates of the row and column vector in the importance degree matrix. If *A*_*ij*_ > 0, then *1/A*_*ij*_ is the importance degree determined when comparing *j* with *i* (*A*_*ij*_ = 1, 2, 3…, 9; *1/ A*_*ij*_ = 1, 1/2, 1/3…, 1/9).

#### Solving the Feature Vector and Maximum Characteristic Root

After constructing the importance degree matrix, we then derived its feature vector and the maximum characteristic root via matrix transformation. The weighted values (from the element of the variable layer to the element of the intermediate layer, or the element of the intermediate layer to the element of the target layer) were derived from the element of the normalized feature vector (Kayet et al., 2018).

#### Consistency Evaluation

The consistency of the odds ratios (obtained from the literature) was converted to the importance degree in the importance degree matrix and evaluated using the consistency index (*CI*) and random consistency ratio *(RCR*). The CI was determined by the formula shown in Equation (1).

(1) $$\begin{array}{l} CI=\frac{MCR-n}{n-1} \end{array}$$

In Equation (1), *MCR* represents the feature vector, and *n* represents the order of the matrix.

A *CI* equal to 0 represented the complete consistency, while a *CI* close to 0 represented the satisfactory consistency of the importance degree matrix. As *CI* increased, the inconsistent odds ratios that were apparent in the literature became more serious once converted and incorporated into the importance degree matrix. The RCR was calculated as shown in Equation (2).

(2) $$\begin{array}{l} RCR=\frac{CI}{K} \end{array}$$

In Equation (2), *K* represents the constant related to *n*. For example, when *n* was 3, 4, 5, and 6, the corresponding *K* value was 0.58, 0.96, 1.12, and 1.24, respectively.

A *RCR* ≤ 0.1 indicated good consistency with regard to the odds ratio obtained from the literature when converted into importance degrees. Otherwise, we needed to re-adjust the importance degree in the importance degree matrix.

The normalized feature vector of the importance degree matrix was the weighted values of the elements among layers. The total weighted values of the elements from the variable layer to the target layer originated from the multiplication of the weighted values through the variable layer to the intermediate layer.

### A Scoring Form Based on the Total Weighted Values for the Elements

The weighted values of the elements in the hierarchical structure to predict FFR were excessive. Consequently, it was inconvenient for the cardiologists to identify which patients should undergo FFR. Therefore, to provide a convenient application for cardiologists, we proposed a scoring form that included the elements with the highest total weighted values within the seven elements selected for FFR prediction.

A scoring form was created using the seven selected elements, and the scores were derived from the weighted values. The total score available on the scoring form was 10 points, such that the total score of the selected elements for a patient represented the final score. The higher the final score, the lower the FFR in the coronary artery ≤ 0.8.

### Study Population

A total of 272 consecutive patients attending the Beijing Anzhen Hospital of the Capital Medical University were enrolled between December 2017 and April 2019. Clinical information was available for all these patients, and all underwent coronary arteriography, transthoracic echocardiography, and invasive FFR measurement. Patients were excluded if they had unstable angina, allergy to contrast agents and vasodilators, multivascular stenosis disease, valvular disease, microcirculation disturbance, a history of myocardial infarction, coronary artery bypass grafting, and PCI. Finally, 253 consecutive patients with single-vessel stenosis were enrolled in the study.

#### Transthoracic Echocardiography Analysis

The aortic velocity (AV) of all the 253 patients with available echocardiographic data was measured by an experienced echocardiographic doctor. The left ventricular ejection fraction (LVEF) and left myocardial mass (LVM) were calculated by an empirical formula based on the structure of the heart (D'Andrea et al., 2012).

#### The Analysis of Angiographic and Physiological Measurements

Coronary angiography was performed using standard techniques to confirm the DS, location of stenosis (LS), type of coronary artery distribution (TCAD), and collateral circulation (CC). To induce maximum hyperemia, intravenous adenosine (140 μg/kg/min) was administrated through the central vein. Next, we obtained the pressure waveform of the aortic pressure (P_a_) and distal arterial pressure (P_d_) under maximum hyperemia and calculated the FFR as the ratio of the mean P_d_ to the mean P_a_ (Zhang et al., 2012; Taylor et al., 2013).

#### Statistical Analysis

Data analyses involved clinical statistics, coronary angiography, and transthoracic echocardiography. Continuous variables are presented as means, while categorical variables are presented as numbers and percentages.

To evaluate the performance of the newly developed method, we used the new tool to predict FFR in 253 consecutive patients with coronary stenosis and then compared these data with those derived from the existing methods and invasive FFR.

Next, we calculated the area under the receiver-operating characteristic curves (AUC) with a 95% confidence interval (CI) to evaluate the accuracy of FFR predication by comparing previous methods with the scoring form. The results of this analysis were then used to determine the critical value of the final score to obtain the metrics of sensitivity, specificity, positive predictive value (PPV), and negative predictive value (NPV), with 95% CI by comparing with the results derived from invasive FFR.

## Results

We retrieved 1,652 articles from the existing literature by screening the PubMed and Web of Science databases. We excluded the articles that were duplicated between the two databases and those published prior to 2000. We also excluded the review articles or articles with inconsistent research content. Finally, eight articles were identified which passed the NOS system inspection and adopted the odds ratio for risk factors, as shown in Table 1.

**Table 1:** The distribution of the included eight pieces of literature.

**First author**	**Cases size**	**Cases source**	**Risk factors**	**NOS score**
Aoi et al. (2020)	423	Medical center	Age, DS	7
Borren et al. (2017)	260	Hospital	Age, gender, BMI, LS	9
Kurtul and Ozturk (2017)	2,286	Hospital	CC, age, LVEF	8
Marco et al. (2017)	2,885	Hospital	Age, HR, LVM	8
Megna et al. (2019)	8,816	Institute	Age, gender	8
Safak et al. (2019)	170	Hospital	LVEF, AV	6
Vidalpetiot et al. (2018)	22,672	Hospital	DP, SP	6
Wang et al. (2019)	269	Hospital	TCAD	7

**Figure 1:**
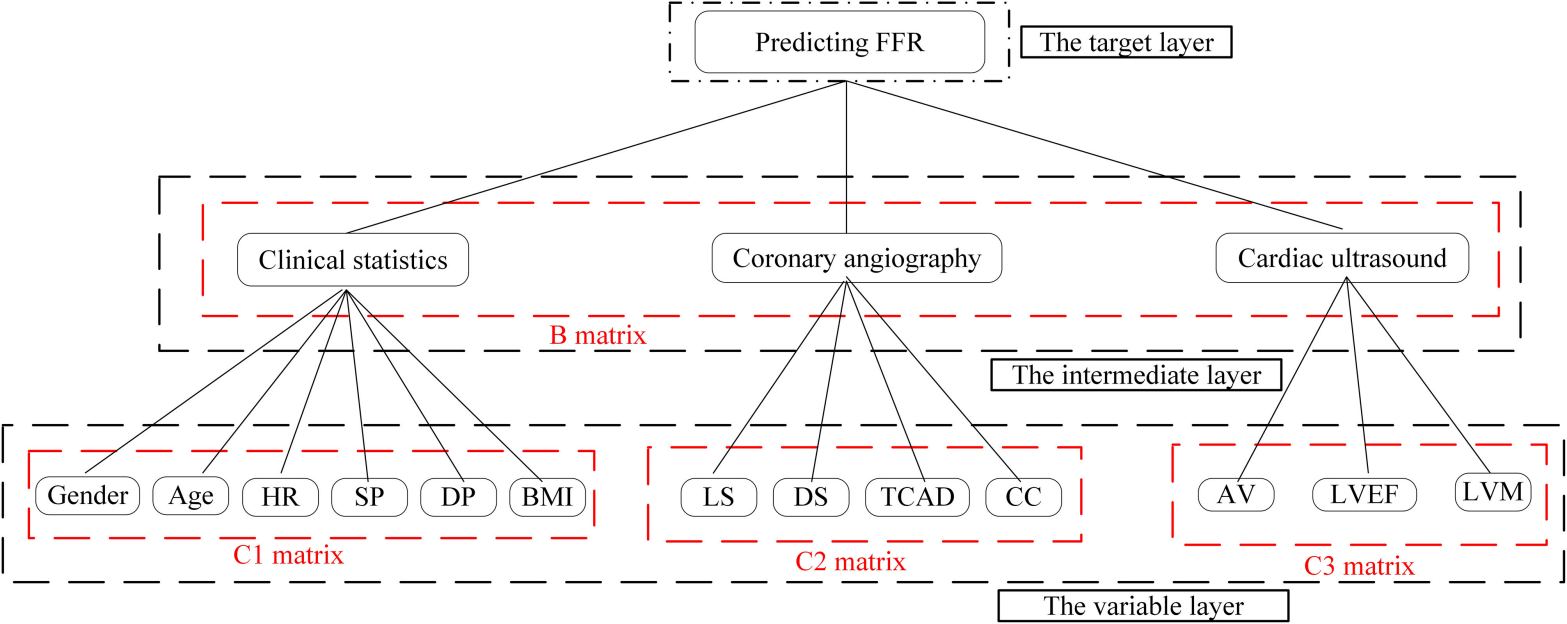
Hierarchical structure about the predicting fractional flow reserve (FFR).

Next, we used the results of our literature analysis to construct a hierarchical structure for predicting FFR. The target layer included the predicting FFR. The intermediate layer included clinical statistics, coronary angiography, and cardiac ultrasound. The variable layer included gender, age, HR, systolic pressure (SP), diastolic pressure (DP), body mass index (BMI), LS, DS, TCAD, CC, AV, LVEF, and LVM, as shown in Figure 1.

**Figure 2:**
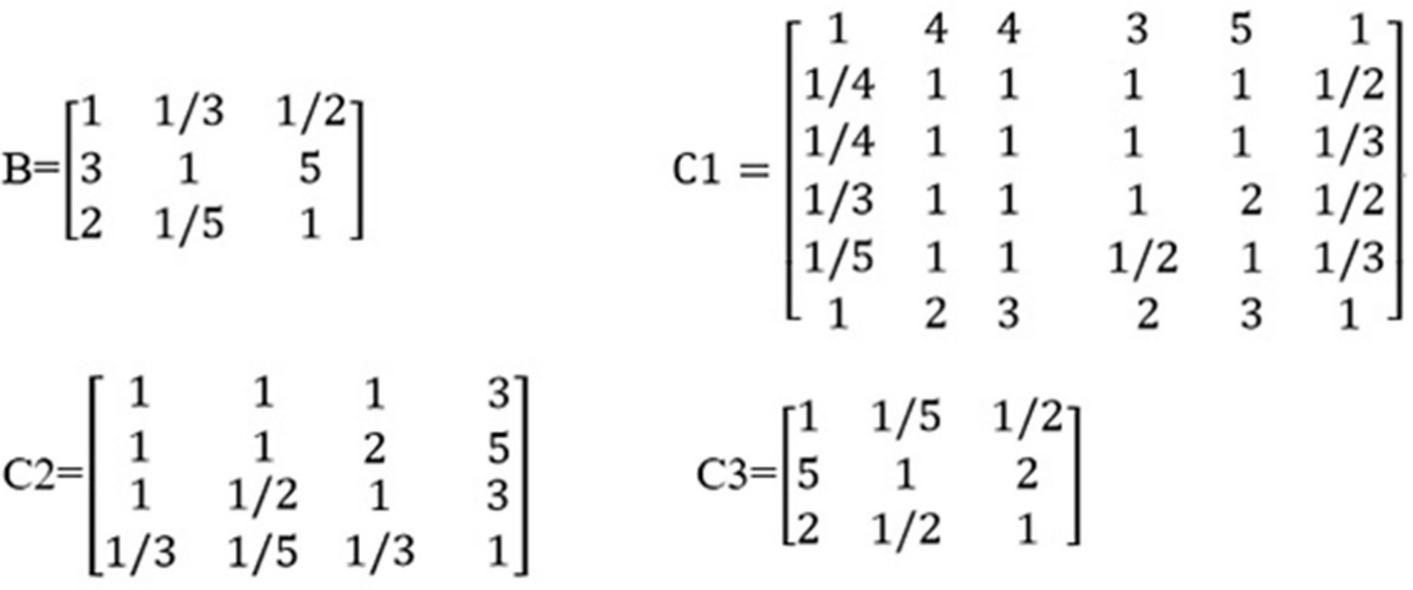
The distribution of the importance degree matrix.

Based on the odds ratios of the elements, we constructed an importance degree matrix (B, C1, C2, and C3) of the comparison between every two elements at the intermediate layer and the variable layer was constructed, as shown in Figure 2.

The matrix B represented the importance degree matrix from the elements of the intermediate layer to the target layer, while the matrices C1, C2, and C3 represented the importance degree matrix from the elements of the variable layer to the intermediate layer. At each of the matrices (B, C1, C2, and C3), the diagonal of the importance degree matrix was 1. This represented that the clinical statistics, coronary angiography, and cardiac ultrasound, as compared with itself, the elements of the gender, age, HR, SP, DP, and BMI compared with itself, LS, DS, TCAD, and CC compared with itself, AV, LVEF, and LVM compared with itself were equally important. In matrix B, numbers 3 and 5 showed that coronary angiography was slightly more important than the clinical statistics and clearly more important than the cardiac ultrasound. In matrix C1, numbers 1/3 and 1/5 included in the first column vector demonstrate that the SP was slightly less important and significantly weaker than the gender.

Table 2 shows that the weighting of each element in one layer to the element in the upper layer was derived from the normalized feature vector of the importance degree matrix. For example, the weighted value of 0.51 represented the degree of influence from the coronary angiography to the predicting FFR in the feature vector of matrix B, 0.35 represented the degree of influence from the factor of the gender to the clinical statistics in the feature vector of matrix C1, 0.387 represented the degree of influence from the DS to the coronary angiography in the feature vector of matrix C2, and 0.59 represented the degree of influence from the LVEF to the cardiac ultrasound in the feature vector of matrix C3.

**Table 2:** The distribution of weight values among the elements of the layers.

**Importance degree matrix**	**Weight value**
B	0.32, 0.51, 0.17
C1	0.35, 0.1, 0.093, 0.11, 0.091, 0.256
C2	0.288, 0.387, 0.242, 0.083
C3	0.13, 0.59, 0.28

Table 3 shows that the resulting CI was close to 0 and RCR was <0.1. This indicates that the odds ratios in the literature showed good consistency when converted and incorporated into the importance degree and importance degree matrix.

**Table 3:** The distribution of importance degree matrix consistency.

**Importance degree matrix**	**MCR**	**CI**	**K**	**RCR**
B	3.05	0.0005	0.58	0.0009
C1	6.11	0.022	1.24	0.0177
C2	4.043	0.014	0.96	0.0146
C3	3.01	0.005	0.58	0.0086

Table 4 shows the total weightings and the impact of each element in the variable layer for the prediction of FFR. DS had the greatest impact on predicting FFR, followed by the elements of LS, TCAD, gender, LVEF, BMI, and so on.

**Table 4:** The sequence of total weight values.

**Target layer**	**Intermediate layer**	**Weight value**	**Variable layer**	**Weight value**	**Sequence**
Predicting FFR	Clinical statistics	0.32	Gender	0.35	4
			Age	0.1	10
			HR	0.093	11
			SP	0.11	12
			DP	0.091	9
			BMI	0.256	6
	Coronary angiography	0.51	LS	0.288	2
			DS	0.387	1
			TCAD	0.242	3
			CC	0.083	8
	Cardiac ultrasound	0.17	AV	0.13	13
			LVEF	0.59	5
			LVM	0.28	7

Table 5 shows that the CI was close to 0 and that the RCR was <0.1, thus indicating that the sequence of the total weight values was reasonable with regard to the impact of an element in the variable layer when predicting FFR.

**Table 5:** The distribution of total weight values consistency.

**CI**	**K**	**RCR**
0.015	0.979	0.0153

The clinical variables of the 253 patients, along with the baseline demographics, are summarized in Table 6. Of these, there were 253 vessels that were assessed with invasive FFR. More than half of the cases in which FFR was measured were men (70.36%). Mean patient age was 59.2 ± 12.5 years, while the mean LVEF value was 57.7 ± 19.3%. The right dominant pattern was the most represented coronary artery distribution (73.91%). Only a small number of patients had CC (3.95%).

**Table 6:** Baseline demographic and clinical characteristics.

**Variable**	**Study population**	
	**Number**	**Percent**
Gender		
Male	178	70.36%
Female	75	29.64%
Age (years)	59.2 ± 12.5	
BMI (kg/m^2^)	23.6 ± 5.3	
HR (beat per minute)	67.5 ± 22.5	
SP (mmHg)	126.4 ± 33.6	
DP (mmHg)	74.1 ± 25.9	
CC	10	3.95%
AV (m/s)	1.27 ± 4.63	
TCAD		
Left dominant pattern	26	10.38%
Right dominant pattern	187	73.91%
Balanced dominant pattern	40	15.81%
LVEF	57.7% ± 19.3%	
LVM (g)	148.56 ± 25.6	

Table 7 presents the distribution of DS and the vessels that had undergone FFR measurement. Most of the measured FFRs were related to the LAD artery (81.03%). More than half of the patients had an intermediate degree of stenosis (51.8%).

**Table 7:** General diameter stenosis (DS) and the vessels with measured fractional flow reserve (FFR).

**Variable**	**Number**	**Percent**
Measured FFR vessels	253	100%
LAD	205	81.03%
LCX	33	13.04%
RCA	15	5.93%
DS (%)		
30–49	13	5.1%
50–69	131	51.8%
70–90	105	41.5%
90 or more	4	1.6%

*LCX, left circumflex artery; RCA, right coronary artery*.

Table 8 shows that a scoring form that includes elements with a higher total weighting within the seven elements can be used to predict FFR. LS and TCAD are the categorical variables. Previous studies showed that male of gender, the LAD stenosis of LS, and a right dominant pattern of TCAD exhibited a significant influence on the prediction of FFR (Sakamoto et al., 2013; Matar et al., 2016; Marco et al., 2017; Wang et al., 2019). Therefore, gender, the LAD stenosis, and the right dominant pattern were separately assigned 1.4, 1.8, and 1.5 points, respectively. Then, DS was assigned 2.4 points, and LVM was assigned 0.7 points. Finally, the mean total score was determined for the 253 single vessels (6.7 ± 2.9 points).

**Table 8:** The distribution of the scoring form.

**Variable**	**Scoring value**
Male gender	1.4
LAD stenosis	1.8
DS ≥ 60%	2.4
Right dominant pattern	1.5
LVEF ≤ 58%	1.2
LVM ≥ 148g	0.7
BMI ≥ 23.5	1
Total score	10

**Figure 3:**
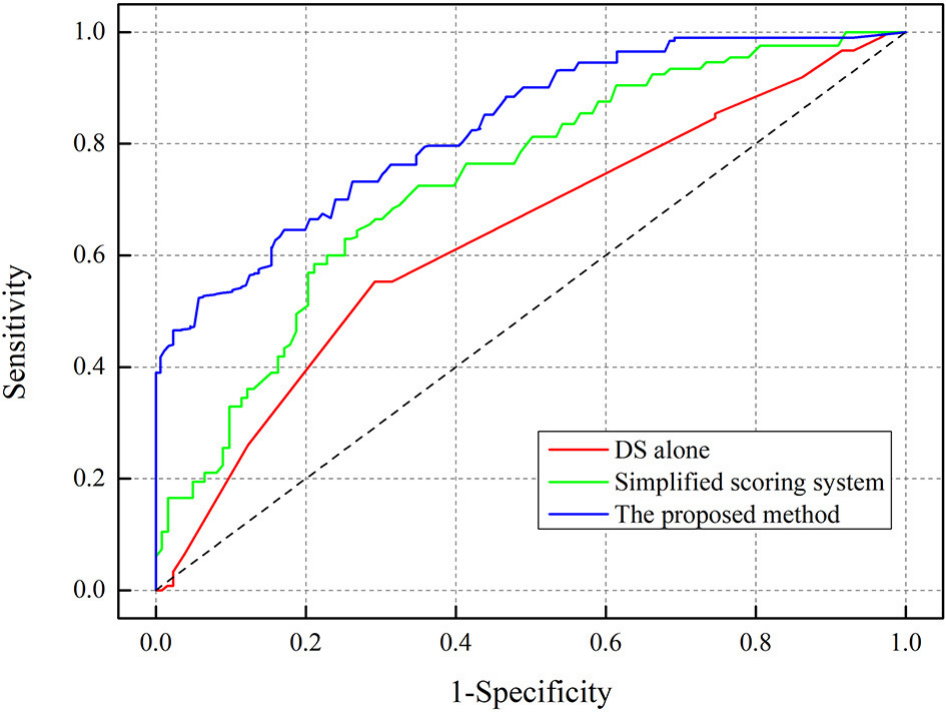
Receiver-operating characteristic (ROC) curve analysis for determining the area under curve (AUC).

The AUC of the scoring form was 84.3% (95% CI: 79.2–89.4%), which was larger than the existing methods, including the use of DS alone of 65.3% (95% CI: 61.5–69.1%), and included a simplified scoring system of 74.8% (95% CI: 68.4–81.2%), as shown in Figure 3. The critical value of the proposed scoring form was ≥6.2 points, which implied that FFR ≤ 0.8. The sensitivity of the FFR prediction was 94.3% (95% CI: 91.9–96.5%), the specificity was 76.2% (95% CI: 69.9–82.5%), the NPV was 93.4% (95% CI: 89.2–97.6%), and the PPV was 78.9% (95% CI: 74.2–83.6%).

## Discussion

In this study, we analyzed 37,781 clinical cases, identified seven independent risk factors, and created a new method to improve the prediction accuracy of FFR. We used IAHP to derive the weight value of each risk factor and constructed the importance degree matrix. Statistical results showed that compared with the clinical diagnosis of the 253 patients (by invasive FFR and coronary angiography), the predictive accuracy of our newly proposed scoring form was 84.3%, and this was higher than the accuracies reported in previous studies (Park et al., 2012; Cho et al., 2014; Curzen et al., 2014; Matar et al., 2016). The main contributors to this study that led to a high prediction accuracy were (1) the identification of seven independent risk factors and (2) the optimized quantitative analysis of the weightings of the risk factors based on odds ratios.

Numerous previous studies have shown that the prediction of FFR had some limitations when DS was used as the only criterion. Many studies showed that the prediction accuracy of FFR using DS was low (≤35%) (Park et al., 2012; Cho et al., 2014; Curzen et al., 2014). Angiography-derived FFR was invasive detection and ignored individual risk factors of patients to predict FFR (Kogame et al., 2020; Suzuki et al., 2020; Ding et al., 2021). With the development of predictive FFR technology, some studies, including case reports, have shown that other risk factors besides DS can also have a significant impact on the prediction of FFR. For example, Taylor et al. (2017) showed that changes in the LVM had a significant impact on FFR changes. In another study, Lee et al. (2015) demonstrated that LAD stenosis was an independent risk factor for the prediction of FFR. Sakamoto et al. (2013) reported an association between the right dominant pattern and FFR prediction based on the extent of the myocardial perfusion area. Gioia et al. (2020) evaluated the significant impact of LVEF changes on changes in the FFR. In addition, clinical statistical studies, based on big data, showed that some basic physiological indicators (such as gender and BMI) can also be regarded as independent risk factors for predicting FFR. On the one hand, men have a larger vessel size and myocardial mass and a lower prevalence of microvascular diseases, than women, and these factors are known to be related to the occurrence of FFR ≤ 0.8 (Kim et al., 2012; Matar et al., 2016; Westergren et al., 2017). On the other hand, as BMI increases, demand of an individual for cardiac blood flow increases, but the coronary blood supply shows a decline, thus leading to an FFR ≤ 0.8 (Matar et al., 2016; Vidalpetiot et al., 2018). However, due to the limitations in clinical data, only a few risk factors were used to establish the prediction systems used in these previous studies. Therefore, a comprehensive and analytical study of all such risk factors was urgently required. Our analysis led to the development of a new method for predicting FFR that exhibited high levels of accuracy.

Numerous previous studies have proved that the weighted calculation of independent risk factors, based on the subjective empirical judgment of the clinicians involved, had obvious limitations (Natsumeda et al., 2015; Wong et al., 2015; Matar et al., 2016). Previous studies have explored the application of a quantitative analysis for the weightings of independent risk factors in order to improve the predictive accuracy for FFR. Biasco et al. (2015) showed that the qualitative analysis of weightings for lesion characteristics and supply area led to improvements in the predictive accuracy of FFR, based on the multivariate analysis of clinical cases. Kang et al. (2013) explained the association between the qualitative analysis of the weightings of gender, age, and anatomy-based structural characteristics; this led to a clear improvement in predictive accuracy for FFR. In another study, Lee et al. (2019) demonstrated that the qualitative analysis of weightings for anatomy-based models, adverse plaque characteristics, and adverse hemodynamic characteristics led to improvements in the predictive accuracy for FFR. Gaur et al. (2016) reported that the qualitative analysis of the weightings for DS, low-density non-calcified plaques, and adverse hemodynamic characteristics had a significant impact on improving the predictive accuracy for FFR. However, these studies ignored the direct or indirect influence of independent risk factors and interactions among the weightings for independent risk factors and the improvement of predictive accuracy for FFR. Predicting FFR using the proposed method optimized the calculation of the weighting of risk factors into a comparison of importance degree based on the odds ratio and was divided into three layers, namely, the target, intermediate, and variable layers, using the method of IAHP. Based on IAHP, FFR was predicted by way of decomposition, comparison, and judgment; the model also featured a systematic and integrated approach. The advantage of using the IAHP tool was the generation of weightings for key elements, including seven independent risk factors, clinical statistics, coronary angiography, and cardiac ultrasound; these factors can all influence the prediction of FFR, either directly or indirectly. Consequently, our method ensured that we considered the relationship between every element and the accurate prediction of FFR. This means that the extent of different effects was quantified in a clear and explicit way. The widespread application of transportation planning also proved that IAHP exhibits high performance with regard to improving the predictive accuracy of optimized protocols (Zhang et al., 2013; Ghorbanzadeh et al., 2018; Moslem et al., 2019). The proposed method involved the quantitative assessment of weightings for seven independent risk factors based on comparisons of weightings for every two independent risk factors by IAHP. The proposed method exhibited improved levels of accuracy for the prediction of FFR when compared with previous methods.

### Limitations and Future Work

Despite the valuable information derived from our newly developed method with regard to improving the predictive accuracy for FFR, several limitations are notable. First, clinical cases were obtained from eight pieces of literature, and it was not good enough. So, we plan to enroll clinical cases from the database of hospitals in our future clinical research. Second, the weighting of independent risk factors including gender, LS, TCAD, and CC had only one value though these independent risk factors had multiple types. For example, the LS can be further divided into proximal stenosis, bifurcation stenosis, and distal stenosis. So, we plan to further optimize the predicted layered structure to calculate the weight of each independent risk factor. Finally, 253 clinical cases that had undergone invasive FFR and coronary angiography were recruited from a single-center database. The diversity of cases may have some deficiencies. Future studies should aim to acquire the diversity of cases from multiple centers.

## Conclusion

In this study, we developed a new method for the prediction of FFR that was based on multiple independent risk factors that were identified from the published literature. We also used IAHP to calculate the relative weightings of these factors so as to improve the predictive accuracy of FFR. Besides the commonly used DS, we should also consider a range of other independent risk factors, including gender, LS, TCAD, LVEF, LVM, and BMI. These risk factors could help to improve the predictive accuracy for FFR. Collectively, the data from this study indicate that the newly developed method can effectively improve the prediction accuracy for FFR.

## Data Availability Statement

The original contributions presented in the study are included in the article/supplementary material, further inquiries can be directed to the corresponding author.

## Ethics Statement

The collection of clinical data and related details of this study were approved by the Institutional Ethics Committee of Beijing Anzhen Hospital (Beijing, China). The collection of clinical data was carried out in accordance with relevant guidelines and regulations. Patient informed consent was waived due to the retrospective nature of the study.

## Author Contributions

HZ was responsible for deciding literature retrieval, data analysis, and paper preparation. HW and YuY screened the pieces of literature. YiY and ZQ assisted in data analysis. JX was responsible for providing clinical data. GL and QH were responsible for language modification. AQ was responsible for supervision. All authors contributed to the article and approved the submitted version.

## Conflict of Interest

The authors declare that the research was conducted in the absence of any commercial or financial relationships that could be construed as a potential conflict of interest.

## Publisher's Note

All claims expressed in this article are solely those of the authors and do not necessarily represent those of their affiliated organizations, or those of the publisher, the editors and the reviewers. Any product that may be evaluated in this article, or claim that may be made by its manufacturer, is not guaranteed or endorsed by the publisher.

## References

[B1] Aoi S. Toklu B. Misumida N. Patel N. Lee W. Fox J.. (2020). Effect of sex difference on discordance between instantaneous wave-free ratio and fractional flow reserve. CRM 9, 1–13. 10.1016/j.carrev.2020.08.013 32839130

[B2] Biasco L. Pedersen F. Lønborg J. Holmvang L. Helqvist S. Saunamäki K.. (2015). Angiographic characteristics of intermediate stenosis of the left anterior descending artery for determination of lesion significance as identified by fractional flow reserve. Am. J. Cardiol. 115, 1475–1480. 10.1016/j.amjcard.2015.02.047 25857401

[B3] Borren N. M. Ottervanger J. P. Reinders M. A. Kedhi E. (2017). Coronary artery stenoses more often overestimated in older patients: angiographic stenosis overestimation in elderly. Int. J. Cardiol. 241, 46–49. 10.1016/j.ijcard.2017.02.134 28318655

[B4] Bruyne B. D. Fearon W. F. Pijls N. H. Barbato E. Tonino P. Piroth Z.. (2014). Fractional flow reserve-guided PCI for stable coronary artery disease. N. Engl. J. Med. 371, 1208–1217. 10.1056/NEJMoa1408758 25176289

[B5] Caroline B. Gianluca P. Mark R. (2018). Fractional flow reserve derived from coronary computed tomography angiography datasets: the Next Frontier in noninvasive assessment of coronary artery disease. Biomed. Res. Int. 4, 1–8. 10.1155/2018/2680430 30276202 PMC6151685

[B6] Chinnaiyan K. M. Akasaka T. Amano T. Bax J. J. Blanke P. Bruyne B. D.. (2017). Rationale, design and goals of the HeartFlow assessing diagnostic value of non-invasive FFRCT in coronary care (ADVANCE) registry. J. Cardiovasc. Comput. Tomogr. 11, 62–67. 10.1016/j.jcct.2016.12.002 28017291

[B7] Cho H. O. Nam C. W. Cho Y. K. Yoon H. J. Park H. S. Kim H.. (2014). Characteristics of function-anatomy mismatch in patients with coronary artery disease. Korean. Circ. J. 44, 394–399. 10.4070/kcj.2014.44.6.394 25469141 PMC4248611

[B8] Curzen N. Rana O. Nicholas Z. Golledge P. Zaman A. Oldroyd K.. (2014). Does routine pressure wire assessment influence management strategy at coronary angiography for diagnosis of chest pain?: the RIPCORD study. Circ. Cardiovasc. Interv. 7, 248–255. 10.1161/CIRCINTERVENTIONS.113.000978 24642999

[B9] D'Andrea A. Nistri S. Castaldo F. Galderisi M. Mele D. Agricola E.. (2012). The relationship between early left ventricular myocardial alterations and reduced coronary flow reserve in non-insulin-dependent diabetic patients with microvascular angina. Int. J. Cardiol. 154, 250–255. 10.1016/j.ijcard.2010.09.044 21035209

[B10] Ding D. Huang J. Westra J. Cohen D. J. Chen Y. Andersen B. K.. (2021). Immediate post-procedural functional assessment of percutaneous coronary intervention: current evidence and future directions. Eur. Heart. J. 5:ehab186. 10.1093/eurheartj/ehab186 33822922

[B11] Gaur S. Øvrehus K. A. Dey D. Leipsic J. Bøtker H. E. Jensen J. M.. (2016). Coronary plaque quantification and fractional flow reserve by coronary computed tomography angiography identify ischaemia-causing lesions. Eur. Heart. J. 37, 1220–1227. 10.1093/eurheartj/ehv690 26763790 PMC4830909

[B12] Geng L. Yuan Y. Du P. Z. Gao L. M. Wang Y. K. Li J. M.. (2021). Association of quantitative flow ratio-derived microcirculatory indices with anatomical-functional discordance in intermediate coronary lesions. Int. J. Cardiovasc. Imaging. 37, 1–11. 10.1007/s10554-021-02292-2 34059977

[B13] Ghorbanzadeh O. Moslem S. Blaschke T. Duleba S. (2018). Sustainable urban transport planning considering different stakeholder groups by an Interval-AHP decision support model. Sustainability 11, 1–18. 10.3390/su11010009

[B14] Gioia G. D. Bruyne B. D. Pellicano M. Bartunek J. Colaiori I. Fiordelisi A.. (2020). Fractional flow reserve in patients with reduced ejection fraction. Eur. Heart. J. 41, 1665–1672. 10.1093/eurheartj/ehz571 31419282

[B15] Han Y. M. Zhou R. D. Geng Z. Q. Bai J. Ma B. Fan J. Z. (2020). A novel data envelopment analysis cross-model integrating interpretative structural model and analytic hierarchy process for energy efficiency evaluation and optimization modeling: application to ethylene industries. J. Clean. Prod. 246, 118965.1–118965.13. 10.1016/j.jclepro.2019.118965

[B16] Kang S. J. Ahn J. M. Han S. Lee J. Y. Kim W. J. Park D. W.. (2013). Sex differences in the visual-functional mismatch between coronary angiography or intravascular ultrasound versus fractional flow reserve. JACC. Cardiovasc. Interv. 6, 562–568. 10.1016/j.jcin.2013.02.016 23787231

[B17] Kayet N. Chakrabarty A. Pathak K. Sahoo S. Dutta T. Hatai B. K. (2018). Comparative analysis of multi-criteria probabilistic FR and AHP models for forest fire risk (FFR) mapping in Melghat Tiger Reserve (MTR) forest. J. Forestry. Res. 31, 565–579. 10.1007/s11676-018-0826-z

[B18] Kharat M. G. Murthy S. Kamble S. J. Raut R. D. Kamble S. S. Kharat M. G. (2019). Fuzzy multi-criteria decision analysis for environmentally conscious solid waste treatment and disposal technology selection. Technol. Soc. 57, 20–29. 10.1016/j.techsoc.2018.12.005

[B19] Kim H. S. Tonino P. A. Bruyne B. D. Yong A. S. Tremmel J. A. Pijls N. H.. (2012). The impact of sex differences on fractional flow reserve-guided percutaneous coronary intervention: a FAME (fractional flow reserve versus angiography for multivessel evaluation) substudy. JACC. Cardiovasc. Interv. 5, 1037–1042. 10.1016/j.jcin.2012.06.016 23078733

[B20] Kogame N. Ono M. Kawashima H. Tomaniak M. Hara H. Leipsic J.. (2020). The impact of coronary physiology on contemporary clinical decision making. JACC. Cardiovasc. Interv. 13, 1617–1638. 10.1016/j.jcin.2020.04.040 32703589

[B21] Koo B. K. Erglis A. Doh J. H. Daniels D. V. Jegere S. Kim H. S.. (2011). Diagnosis of ischemia-causing coronary stenosis by noninvasive fractional flow reserve computed from coronary computed tomographic angiograms results from the prospective multicenter DISCOVER-FLOW (Diagnosis of ischemia-causing stenosis obtained via noninvasive fractional flow reserve) study. J. Am. Coll. Cardiol. 58, 1989–1997. 10.1016/j.jacc.2011.06.066 22032711

[B22] Kurtul A. Ozturk S. (2017). Prognostic value of coronary collaterals in patients with acute coronary syndromes. Coron. Artery. Dis. 28, 406–412. 10.1097/MCA.0000000000000500 28617303

[B23] Lee J. M. Choi G. Koo B. K. Hwang D. Park J. Zhang J. L.. (2019). Identification of high-risk plaques destined to cause acute coronary syndrome using coronary computed tomographic angiography and computational fluid dynamics. JACC. Cardiovasc. Imaging 6, 1032–1043. 10.1016/j.jcmg.2018.01.023 29550316

[B24] Lee J. M. Layland J. Jung J. H. Lee H. J. Echavarria-Pinto M. Watkins S.. (2015). Integrated physiologic assessment of ischemic heart disease in real-world practice using index of microcirculatory resistance and fractional flow reserve. Circ. Cardiovasc. Interv. 8, 1–8. 10.1161/CIRCINTERVENTIONS.115.002857 26499500

[B25] Li G. Y. Wang H. R. Zhang M. Z. Tupin S. Qiao A. K. Liu Y. J.. (2021). Prediction of 3D cardiovascular hemodynamics before and after coronary artery bypass surgery via deep learning. Commun. Biol. 4, 1–12. 10.1038/s42003-020-01638-1 33483602 PMC7822810

[B26] Lu M. T. Ferencik M. Roberts R. S. Lee K. L. Ivanov A. Adami E.. (2017). Noninvasive FFR derived from coronary CT angiography: management and outcomes in the PROMISE trial. JACC. Cardiovasc. Imaging 10, 1350–1358. 10.1016/j.jcmg.2016.11.024 28412436 PMC5632098

[B27] Marco M. D. Gerdts E. Mancusi C. Roman M. J. Lønnebakken M. T. Lee E. T.. (2017). Influence of left ventricular stroke volume on Incident heart failure in a population with preserved ejection fraction (from the Strong Heart Study). J. Am. Coll. Cardiol. 119, 1047–1052. 10.1016/j.amjcard.2016.12.011 28159195 PMC5348268

[B28] Matar F. A. Falasiri S. Glover C. B. Khaliq A. Leung C. C. Mroue J.. (2016). When should fractional flow reserve be performed to assess the significance of borderline coronary artery lesions: derivation of a simplified scoring system. Int. J. Cardiol. 222, 606–610. 10.1016/j.ijcard.2016.07.171 27517648

[B29] Megna R. Zampella E. Assante R. Nappi C. Gaudieri V. Mannarino T.. (2019). Temporal trends of abnormal myocardial perfusion imaging in a cohort of Italian subjects: relation with cardiovascular risk factors. J. Nucl. Cardiol. 27, 2167–2177. 10.1007/s12350-019-01630-1 30734219

[B30] Mokarram M. Saber A. Mohammadizadeh P. Abdolali A. (2020). Determination of artifcial recharge location using analytic hierarchy process and Dempster–Shafer theory. Environ. Earth. Sci. 79, 1–15. 10.1007/s12665-020-08994-5

[B31] Moslem S. Ghorbanzadeh O. Blaschke T. Duleba S. (2019). Analysing stakeholder consensus for a sustainable transport development decision by the Fuzzy AHP and Interval AHP. Sustainability 11, 1–22. 10.3390/su11123271

[B32] Nakamura M. Yamagishi M. Ueno T. Hara K. Ishiwata S. Itoh T.. (2014). Prevalence of visual–functional mismatch regarding coronary artery stenosis in the CVIT-DEFER registry. Cardiovasc. Interv. Ther. 29, 300–308. 10.1007/s12928-014-0259-3 24664513

[B33] Natsumeda M. Nakazawa G. Murakami T. Torii S. Ijichi T. Ohno Y.. (2015). Coronary angiographic characteristics that influence fractional flow reserve. Circ. J. 79, 802–807. 10.1253/circj.CJ-14-0931 25739718

[B34] Nørgaard B. L. Leipsic J. Gaur S. Seneviratne S. Ko S. S. Ito H.. (2014). Diagnostic performance of noninvasive fractional flow reserve derived from coronary computed tomography angiography in suspected coronary artery disease: the NXT trial (analysis of coronary blood flow using CT angiography: next steps). J. Am. Coll. Cardiol. 63, 1145–1155. 10.1016/j.jacc.2013.11.043 24486266

[B35] Park S. J. Kang S. J. Ahn J. M. Shim E. B. Kim Y. T. Yun S. C.. (2012). Visual-functional mismatch between coronary angiography and fractional flow reserve. JACC. Cardiovasc. Interv. 10, 1029–1036. 10.1016/j.jcin.2012.07.007 23078732

[B36] Pham N. M. Do V. V. Lee A. H. (2019). Polyphenol-rich foods and risk of gestational diabetes: a systematic review and meta-analysis. Eur. J. Clin. Nutr. 73, 647–656. 10.1038/s41430-018-0218-7 29941912

[B37] Pijls N. H. Schaardenburgh P. V. Manoharan G. Boersma E. Bech J. W. Van't Veer M.. (2007). Percutaneous coronary intervention of functionally nonsignificant stenosis: 5-year follow-up of the DEFER study. J. Am. Coll. Cardiol. 49, 2105–2111 10.1016/j.jacc.2007.01.087 17531660

[B38] Safak E. Ince H. Gkouvatsou L. Schultheiss H. P. Ortak J. Caglayan E.. (2019). Pacing-induced cardiomyopathy in chronic right ventricular apical pacing: a midterm follow-up study. Eur. J. Med. Res. 24, 1–5. 10.1186/s40001-019-0386-5 31331388 PMC6643303

[B39] Sakamoto S. Takahashi S. Coskun A. U. Papafaklis M. Takahashi A. Saito P. H.. (2013). Relation of distribution of coronary blood flow volume to coronary artery dominance. J. Am. Coll. Cardiol. 111, 1420–1424. 10.1016/j.amjcard.2013.01.290 23540543

[B40] Suzuki N. Asano T. Nakazawa G. Aoki J. Tanabe K. Hibi K.. (2020). Clinical expert consensus document on quantitative coronary angiography from the Japanese Association of Cardiovascular Intervention and Therapeutics. Cardiovasc. Interv. Ther. 35, 105–116. 10.1007/s12928-020-00653-7 32125622 PMC7105443

[B41] Taylor C. A. Fonte T. A. Min J. K. (2013). Computational fluid dynamics applied to cardiac computed tomography for noninvasive quantification of fractional flow reserve: scientific basis. J. Am. Coll. Cardiol. 61, 2233–2241. 10.1016/j.jacc.2012.11.083 23562923

[B42] Taylor C. A. Gaur S. Leipsic J. Achenbach S. Berman D. S. Jensen J. M.. (2017). Effect of the ratio of coronary arterial lumen volume to left ventricle myocardial mass derived from coronary CT angiography on fractional flow reserve. J. Cardiovasc. Comput. 7, 429–436. 10.1016/j.jcct.2017.08.001 28789941

[B43] Tian X. Chen C. Y. Ma L. Y. Wei R. Li M. Wang X. Q.. (2020). Efficacy and safety of rituximab in relapsing-remitting multiple sclerosis: a systematic review and meta-analysis. J. Neuroimmunol. 347, 1–9. 10.1016/j.jneuroim.2020.577317 32731048

[B44] Tonino P. A. Fearon W. F. Bruyne B. D. Oldroyd K. G. Leesar M. A. Ver Lee P. N.. (2010). Angiographic versus functional severity of coronary artery stenoses in the FAME study fractional flow reserve versus angiography in multivessel evaluation. J. Am. Coll. Cardiol. 55, 2816–2821. 10.1016/j.jacc.2009.11.096 20579537

[B45] Tu S. X. Westra J. Yang J. Q. Von Birgelen C. Ferrara A. Pellicano M.. (2016). Diagnostic accuracy of fast computational approaches to derive fractional flow reserve from diagnostic coronary angiography: The International Multicenter FAVOR Pilot Study. JACC. Cardiovasc. Interv. 19, 2024–2035. 10.1016/j.jcin.2016.07.013 27712739

[B46] Vidalpetiot E. Ford I. Greenlaw N. Ferrari R. Fox K. M. Tardif J. C.. (2018). Cardiovascular event rates and mortality according to achieved systolic and diastolic blood pressure in patients with stable coronary artery disease: an international cohort study. Lancet 388, 2142–2152. 10.1016/S0140-6736(16)31326-5 27590221

[B47] Wang L. Li J. M. Gao Y. Li R. H. Zhang J. J. Su D.. (2019). Association between coronary dominance and acute inferior myocardial infarction: A matched, case-control study. BMC. Cardiovasc. Disord. 19, 1–7. 10.1186/s12872-019-1007-5 30717670 PMC6360684

[B48] Westergren H. U. Michaëlsson E. Blomster J. I. Miliotis T. Svedlund S. Gan L. M. (2017). Determinants of coronary flow reserve in non-diabetic patients with chest pain without myocardial perfusion defects. PLoS ONE 12, 1–16. 10.1371/journal.pone.0176511 28448601 PMC5407821

[B49] Westra J. Andersen B. K. Campo G. Matsuo H. Koltowski L. Eftekhari A.. (2018). Diagnostic performance of in procedure angiography derived quantitative flow reserve compared to pressure-derived fractional flow reserve: the FAVOR II Europe-Japan Study. J. Am. Heart. Assoc. 7:e009603. 10.1161/JAHA.118.009603 29980523 PMC6064860

[B50] Wong D. T. Narayan O. Ko B. S. Leong D. P. Seneviratne S. Potter E. L.. (2015). A novel coronary angiography index (DILEMMA score) for prediction of functionally significant coronary artery stenoses assessed by fractional flow reserve: a novel coronary angiography index. Am. Heart. J. 169, 564–571. 10.1016/j.ahj.2014.11.017 25819864

[B51] Yang D. H. Kim Y. H. Roh J. H. Kang J. W. Ahn J. M. Kweon J.. (2017). Diagnostic performance of on-site CT-derived fractional flow reserve versus CT perfusion. Eur. Heart. J-Card. Img. 18, 432–440. 10.1093/ehjci/jew094 27354345

[B52] Zhang S. Bo S. Lei Y. Wang C. (2013). Risk identification on hydropower project using the IAHP and extension of TOPSIS methods under interval-valued fuzzy environment. Nat. Hazards 65, 359–373. 10.1007/s11069-012-0367-2

[B53] Zhang Z. Takarada S. Molloi S. (2012). Quantification of absolute coronary flow reserve and relative fractional flow reserve in a swine animal model using angiographic image data. Am. J. Physiol. Heart. Circ. Physiol. 303, 1–10. 10.1152/ajpheart.00153.2012 22661513 PMC3423159

